# Implementation of Evidence-Based Practices in Intergenerational Programming: A Scoping Review

**DOI:** 10.1093/geroni/igaa057.2603

**Published:** 2020-12-16

**Authors:** Shannon Jarrott, Rachel Scrivano, Nancy Mendoza, Cherrie Park

**Affiliations:** The Ohio State University, Columbus, Ohio, United States

## Abstract

Intergenerational programs facilitate mutually beneficial interactions between youth and older adults, achieving an array of outcomes. With few exceptions, implementation factors rarely figure into outcome analyses, though researchers frequently gather data on factors influencing outcomes. The resulting practice-evidence gap may deter wide-spread adoption of intergenerational programming. We conducted a scoping review of 35 peer-reviewed articles (2000-2019) to map key concepts and evidence sources of empirically-supported practices impacting intergenerational program outcomes. A scoping review is appropriate when an area is complex, like intergenerational programs that incorporate diverse participants, content, and goals. Primarily qualitative studies involved programs equally likely to involve young, school-age, or post-secondary age youth and independent or frail older adults. Half had sample sizes under 50, frequently measuring both age groups. Implementation practices included co-learning and sharing personal stories. Program content (e.g., technology) and greater exposure amplified outcomes. Rigorous implementation research is needed to advance evidence-based intergenerational practice.

